# Association of Long‐Term Oral β‐Blocker Use With Cardiac Troponin I and B‐Type Natriuretic Peptide in Septic Myocardial Injury: A Retrospective Cohort Study

**DOI:** 10.1002/hsr2.71573

**Published:** 2025-11-26

**Authors:** Zheng Yang, Aihua Jiang, Yunlong Zhu, Kaiqi Cao, Jianbiao Meng, Fang Han, Chun Yang

**Affiliations:** ^1^ Emergency Department Tongde Hospital of Zhejiang Province Affiliated to Zhejiang Chinese Medical University Hangzhou China; ^2^ Intensive Care Unit Tongde Hospital of Zhejiang Province Affiliated to Zhejiang Chinese Medical University Hangzhou China; ^3^ Department of Emergency Medicine Emergency and Critical Care Center, Zhejiang Provincial People's Hospital (Affiliated People's Hospital), Hangzhou Medical College Hangzhou Zhejiang China

**Keywords:** β‐blockers, BNP, critical care, cTnI, retrospective study, septic myocardial injury

## Abstract

**Background and Aims:**

Septic myocardial injury, characterized by elevated levels of cardiac troponin I (cTnI) and B‐type natriuretic peptide (BNP), is associated with increased mortality in sepsis. This study aimed to evaluate whether chronic oral β‐blocker use is associated with reduced cardiac biomarker levels and improved short‐term outcomes.

**Methods:**

We conducted a retrospective cohort study of 289 patients diagnosed with septic myocardial injury admitted to the ICU/EICU from January 2015 to June 2020. Patients were stratified into chronic β‐blocker users (*n* = 102) and non‐users (*n* = 187). Associations between β‐blocker use and serum cTnI and BNP levels were analyzed using univariate and multivariate linear regression, adjusting for age, severity scores (SOFA, APACHE II), infection site, and vasopressor use. Secondary outcomes included 28‐day mortality and ICU length of stay. Subgroup analyses were performed by age and infection site.

**Results:**

Chronic β‐blocker use was independently associated with significantly lower serum levels of cTnI (0.19 vs. 0.29 μg/L; *β* = −0.07; 95% CI: −0.12 to −0.02; *p* < 0.001) and BNP (627 vs. 690 ng/L; *β* = −36.6; 95% CI: −69.6 to −3.5; *p* = 0.03). However, no significant differences were observed in 28‐day mortality or ICU length of stay. Subgroup analysis revealed greater biomarker reductions in patients under 60 years old and those with pulmonary infections, though sample sizes were limited.

**Conclusion:**

Long‐term β‐blocker therapy is associated with lower levels of cardiac injury biomarkers in patients with septic myocardial injury. Subgroup analyses revealed greater reductions in younger patients and those with pulmonary infections, though these findings are exploratory. However, these biochemical improvements did not translate into short‐term survival benefits. Prospective trials are needed to determine whether myocardial protection translates into clinical benefit.

## 1 Introduction

Sepsis is a life‐threatening syndrome characterized by a dysregulated host response to infection, leading to multiple organ dysfunction and high mortality rates among critically ill patients [1]. Myocardial injury is a common complication of sepsis, often indicated by elevated levels of cardiac troponin I (cTnI) and B‐type natriuretic peptide (BNP) [2]. This cardiac involvement significantly contributes to poor prognosis in septic patients. The underlying pathophysiology is multifactorial, involving oxidative stress, inflammation, calcium overload, and apoptosis [3]. Timely detection and management of myocardial damage are crucial to improving outcomes in this population [2].

β‐blockers, commonly prescribed for chronic cardiovascular diseases, exert protective effects on the heart by antagonizing β‐adrenergic receptors [4, 5]. This results in reduced heart rate, myocardial contractility, and oxygen demand through the suppression of catecholamines such as adrenaline and noradrenaline [3, 6]. Emerging evidence also suggests that β‐blockers may exert anti‐inflammatory and immunomodulatory effects, which could be beneficial in sepsis management [7].

In our previous study, we reported that long‐term oral β‐blocker use was associated with reduced levels of cTnI and BNP in patients with sepsis‐related myocardial injury [8]. However, that analysis was limited to basic group comparisons and did not fully adjust for potential confounders, leaving uncertainty regarding the independent effect of β‐blocker therapy.

Building upon these preliminary findings, the present study aims to further explore the association between chronic oral β‐blocker use and myocardial injury in septic patients. Using the same data set, we implemented a more comprehensive statistical approach, including univariate analysis, multivariate regression, and subgroup analyses. These methods allow for better adjustment of clinical and demographic variables and provide a more robust evaluation of β‐blockers' potential cardioprotective role in the context of sepsis. Through this refined methodology, we aim to offer deeper insights that may inform future therapeutic strategies and clinical decision‐making.

## 2 Methods

### 2.1 Data Source

The study utilized data extracted from the electronic medical records system of Tonde Hospital of Zhejiang Province, focusing on the EICU and ICU databases. These databases cover patient admissions from January 2015 to June 2020, offering a wealth of information including demographic profiles, medical histories, laboratory findings, treatments, and clinical outcomes. This data set served as the basis for our retrospective investigation into patients with Septic Myocardial Injury, examining the impact of extended oral β‐blocker use on their cardiac biomarkers.

We applied the following inclusion and exclusion criteria for patient selection:

Inclusion Criteria

1. Patients diagnosed with septic myocardial injury, defined as [9, 10]: (1) proven or suspected infection; (2) serum cTnI ≥ 0.04 μg/L within 24 h of sepsis onset; (3) Exclusion of alternative causes of myocardial injury (e.g., acute coronary syndrome, heart failure).
2. Availability of complete data on β‐blocker usage and relevant cardiac biomarkers (serum cTnI and BNP levels).
3. Adult patients (≥ 18 years) were included.
4. Patients were admitted to the EICU or ICU within 24 h of the onset of sepsis symptoms.

Exclusion Criteria

1. Patients with incomplete data on key variables, including missing data on β‐blocker usage, cTnI, or BNP levels.
2. Patients with other pre‐existing cardiovascular diseases that might confound the assessment of myocardial injury, such as coronary heart disease, dilated cardiomyopathy, hypertrophic cardiomyopathy, or valvular heart disease.
3. Patients with a history of cardiac surgery or percutaneous coronary intervention.
4. Patients with severe liver or kidney dysfunction that might affect drug metabolism and cardiac biomarker levels.
5. Patients with a history of long‐term use of other medications that might influence myocardial injury, such as steroids or non‐β‐blocker antiarrhythmic drugs.
6. Patients with a history of malignancy or immune system diseases that might affect the interpretation of study results.
7. Patients who died within 24 h of admission to the EICU or ICU, as adequate assessment of cardiac biomarkers and β‐blocker effects would not be feasible within this short period.

### 2.2 Study Population and Missing Value Handling

Our cohort consisted of 289 patients diagnosed with this condition within a specified timeframe, selected based on strict inclusion and exclusion criteria. Inclusion criteria required patients to meet the diagnostic criteria and have available data on β‐blocker usage and relevant cardiac biomarkers. Patients with incomplete data on key variables were excluded to ensure the reliability of the analysis, resulting in approximately 7.5% missing data. To address this, we utilized multiple imputation, a statistical technique that replaces missing values with multiple plausible sets based on observed data patterns. This method maintains statistical power, reduces bias from incomplete data sets, and enhances the validity of our findings.

### 2.3 Exposure Definition: β‐Blocker Use, Cardiac Biomarkers, and Covariates

Our primary exposure was prolonged oral β‐blocker therapy, defined as daily use for at least 3 months before the diagnosis of septic myocardial injury. We recorded preadmission indications for β‐blocker use—such as hypertension, atrial fibrillation, hyperthyroidism, and essential tremor/migraine—based on patient history and electronic health records. The most commonly prescribed agents were metoprolol (both immediate‐ and sustained‐release) and bisoprolol, all cardio‐selective β₁‐blockers. Treatment duration exceeded 3 months for all patients. Medication adherence was inferred from consistent refill history and inpatient medication reconciliation. However, precise dosage data were not available. We also lacked data on concurrent long‐term medications (e.g., ACEI/ARBs, calcium channel blockers, statins), which may act as unadjusted confounders.

Outcome measures included serum levels of cTnI and BNP, critical markers of myocardial injury. We accounted for a comprehensive set of potential confounders in our univariate and multivariate analyses: demographics (age, sex), clinical signs (heart rate, blood pressure), laboratory indices (AST, ALT, GGT, uric acid, fasting glucose, total cholesterol, LDL, lactate), severity scores (SOFA, APACHE II), infection site, and vasopressor use. All blood tests were conducted within the first 24 h of ICU admission. By integrating these covariates in our analysis, we aimed to clarify the independent association between β‐blocker use and cardiac biomarker changes in septic myocardial injury.

We structured our reporting according to the STROBE guidelines by clearly defining exposures, outcomes, and potential confounders—an essential best practice for observational research [11]. However, as this is an observational study, we remain mindful of confounding by indication, in which the clinical reasons for prescribing β‐blockers may themselves influence outcomes [12].

### 2.4 Statistical Analysis

The study's statistical analysis was carried out in four phases to thoroughly investigate the correlation between extended oral β‐blocker usage and serum cTnI and BNP levels in patients with septic myocardial injury. The specific procedures are outlined below.

Initially, patients were categorized into two groups based on their pre‐admission history of long‐term oral β‐blocker usage: those with prior use (“Yes”) and those without (“No”). This stratification facilitated a direct comparison between the two groups concerning their cardiac biomarker levels and other clinical factors.

Univariate analysis was conducted to explore associations between β‐blocker use and cTnI/BNP levels. This initial step yielded preliminary insights into the possible connection between the variables under study, as well as identifying potential factors associated with cardiac biomarker levels. The univariate analysis laid the groundwork for subsequent, more intricate analyses.

Multimodal regression analysis was utilized to explore the independent association between β‐blocker utilization and cardiac biomarker concentrations. These models facilitated the concurrent adjustment for multiple covariates, thereby mitigating the influence of confounding factors. The regression analysis followed a hierarchical progression, commencing with a basic model involving solely the exposure variable (β‐blocker usage), then advancing to a partially adjusted model integrating fundamental demographic variables (age and sex), and culminating in a fully adjusted model encompassing all pertinent covariates. This systematic approach enabled the evaluation of the consistency of the relationship between β‐blocker utilization and cardiac biomarker levels across various adjustment scenarios.

Subgroup analysis was performed to investigate potential variations in the impact of β‐blockers on cardiac biomarkers among distinct patient subgroups. Stratification of patients was based on critical clinical and demographic factors, including age, sex, infection site, and vasopressor utilization. The association between β‐blocker administration and cardiac biomarker levels was evaluated within each subgroup utilizing regression models. This analytical approach facilitated the identification of particular patient subgroups that could potentially experience enhanced or distinct advantages from β‐blocker treatment.

### 2.5 Data Processing and Analysis

The data were processed and analyzed using R statistical software, version 4.4 (released on April 24, 2024), along with Zstats 1.0. software package (www.zstats.net). These tools offered a wide range of functions for data manipulation, analysis, and visualization. A significance threshold of *p* < 0.05 (two‐tailed) was utilized to establish statistical significance, and effect estimates are accompanied by 95% confidence intervals (95% CI). *p*‐values are presented according to standard formatting practices: *p* < 0.001 for values below 0.001; Three decimal places (e.g., *p* = 0.008) for values between 0.001 and 0.01; Two decimal places (e.g., *p* = 0.24) for values ≥ 0.01; *p* > 0.99 where applicable. All analyses were pre‐specified except for subgroup analyses, which were exploratory.

### 2.6 Ethics Approval and Consent to Participate

The research adhered to the ethical guidelines outlined in the World Medical Association Declaration of Helsinki. Ethics clearance was granted by the Ethics Committee of Tongde Hospital, Zhejiang Province (approval number: 2020‐082), and the study was registered as a clinical trial under the designated number [MR‐33‐22‐021455]. All procedures were conducted as per the approved protocol. Due to the retrospective design of the study and the utilization of de‐identified data, the need for informed consent was exempted by the Ethics Committee.

### 2.7 Reporting Standards

We followed CONSORT principles—despite this being an observational study—to promote transparency, completeness, and replicability, particularly by clearly describing study design, statistical methods, and participant flow [13]. In addition, we adhered to STROBE guidelines for observational studies to ensure comprehensive reporting of methods and results.

### 2.8 Data Availability Statement

The data that support the findings of this study are not publicly available due to institutional restrictions and ethical regulations, but are available from the corresponding author upon reasonable request and subject to approval by the institutional ethics committee.

## 3 Results

### 3.1 Selection of Study Subjects and Baseline Characteristics

A total of 289 patients with septic myocardial injury were identified from the EICU and ICU of our institution. After applying the inclusion criteria and ensuring data integrity, all 289 patients were deemed eligible for the final analysis. Following a meticulous screening procedure to ensure data integrity and adherence to study criteria, 289 eligible patients were included in the final analysis. Among these, 187 patients were not receiving long‐term oral β‐blockers and were classified into the non‐β‐blocker cohort, while 102 patients with a history of long‐term oral β‐blocker usage were allocated to the β‐blocker cohort.

Table 1 summarizes the baseline characteristics. The median age was 74 years (IQR: 68–83), with a nearly equal gender distribution. Comparable distributions of age, gender, infection site, and vasopressor usage were observed between the groups receiving β‐blockers and those not. However, significant differences were observed in several parameters. The non‐β‐blocker group exhibited a significantly higher average heart rate (111 vs. 105 bpm, *p* < 0.001) than the β‐blocker group. Additionally, higher levels of cTnI and BNP, indicative of more severe myocardial injury, were observed in the non‐β‐blocker group. Conversely, levels of AST, ALT, GGT, uric acid, fasting glucose, TC, LDL, and Lac, as well as SOFA and APACHE II scores, did not significantly differ between the two groups.

**Table 1:** Baseline characteristics of patients with septic myocardial injury stratified by β‐blocker use.

Variables	Total (*n* = 289)	No‐β‐blocker agent (*n* = 187)	β‐blocker agent (*n* = 102)	Statistic	*p* value
Gender, *n* (%)				*χ*² = 3.23	0.07
Male	151 (52.25)	105 (56.15)	46 (45.10)		
Female	138 (47.75)	82 (43.85)	56 (54.90)		
Age (years), M (Q₁, Q₃)	74.00 (68.00, 83.00)	77.00 (70.00, 83.00)	73.00 (61.00, 82.00)	*Z* = −1.88	0.06
Infection site, *n* (%)				*χ*² = 1.13	0.29
Pulmonary	225 (77.85)	142 (75.94)	83 (81.37)		
Others*	64 (22.15)	45 (24.06)	19 (18.63)		
Vasoactive agent, *n* (%)				*χ*² = 0.46	0.50
No	121 (41.87)	81 (43.32)	40 (39.22)		
Yes	168 (58.13)	106 (56.68)	62 (60.78)		
HR (bpm), M (Q₁, Q₃)	109.00 (103.00, 115.00)	111.00 (104.00, 115.00)	105.00 (102.00, 111.00)	*Z* = −3.61	< 0.001
MAP (mmHg), M (Q₁, Q₃)	70.00 (67.00, 72.00)	69.00 (67.00, 72.00)	70.00 (68.00, 72.00)	*Z* = −1.54	0.12
ScvO₂, M (Q₁, Q₃)	0.73 (0.71, 0.75)	0.73 (0.71, 0.76)	0.72 (0.71, 0.74)	*Z* = −1.56	0.12
Lac (mmol/L), M (Q₁, Q₃)	3.50 (2.60, 4.40)	3.50 (2.60, 4.45)	3.50 (2.60, 4.40)	*Z* = −0.23	0.82
cTnI (μg/L), M (Q₁, Q₃)	0.23 (0.12, 0.41)	0.29 (0.15, 0.45)	0.19 (0.11, 0.33)	*Z* = −3.26	< 0.001
CKMB (μg/L), M (Q₁, Q₃)	4.32 (3.23, 6.34)	4.32 (3.22, 6.34)	4.24 (3.25, 6.50)	*Z* = −0.32	0.75
BNP (ng/L), M (Q₁, Q₃)	656.00 (586.00, 777.00)	690.00 (590.00, 791.00)	627.00 (586.00, 715.50)	*Z* = −2.01	0.04
E/A, Mean ± SD	0.80 ± 0.23	0.79 ± 0.24	0.83 ± 0.22	*t* = −1.58	0.12
LVEF (%), M (Q₁, Q₃)	56.84 (53.00, 61.00)	56.84 (53.00, 60.00)	56.50 (53.00, 61.00)	*Z* = −0.87	0.38
SOFA, M (Q₁, Q₃)	8.00 (6.00, 9.00)	8.00 (5.00, 9.00)	8.00 (6.00, 9.00)	*Z* = −1.52	0.13
APACHE II, M (Q₁, Q₃)	18.00 (13.00, 19.00)	18.00 (14.00, 20.00)	15.50 (13.00, 19.00)	*Z* = −2.41	0.02
ICU days, M (Q₁, Q₃)	7.00 (5.00, 11.00)	7.00 (5.00, 11.00)	7.00 (5.00, 11.00)	*Z* = −0.49	0.62
Death in 28 days, *n* (%)				*χ*² = 0.16	0.69
No	217 (75.09)	139 (74.33)	78 (76.47)		
Yes	72 (24.91)	48 (25.67)	24 (23.53)		

Abbreviations: APACHE II, Acute Physiology and Chronic Health Evaluation II; β‐blocker, β‐adrenergic blocker; BNP, brain natriuretic peptide; CKMB, creatine kinase – MB isoenzyme; cTnI, cardiac troponin I; E/A, early diastolic velocity/atrial systolic velocity; HR, heart rate; ICU, intensive care unit; Lac, lactic acid; LVEF, left ventricular ejection fraction; MAP, mean arterial pressure; ScvO₂, central venous oxygen saturation; SOFA, Sequential Organ Failure Assessment.

* Abdominal cavity, urinary system, skin soft tissue, and so forth.

### 3.2 Results of Univariate Analysis

Table 2 summarizes the univariate associations. Extended oral β‐blocker therapy was significantly associated with reduced biomarker levels: a decrease in cTnI (*β* = −0.07; 95% CI: −0.11 to −0.02; *p* < 0.001) and BNP (*β* = −36.56 ng/L; 95% CI: −69.64 to −3.49; *p* = 0.03). Other variables—including sex, age, infection site, and vasopressor use—showed no significant correlation with biomarker changes.

**Table 2:** Univariate analysis of factors associated with cardiac biomarker levels in patients with septic myocardial injury.

Variables	Statistics	cTnI (μg/L)		BNP (ng/L)	
		*β* (95% CI)	*p* value	*β* (95% CI)	*p* value
Gender, *n* (%)					
Male	151 (52.25%)	−0.02 (−0.06 to 0.02)	0.36	0.10 (−31.80 to 32.00)	> 0.99
Female	138 (47.75%)	0.00 (Reference)		0.00 (Reference)	
Age, M (Q₁, Q₃)	74.00 (68.00,83.00)	0.00 (−0.00 to 0.00)	0.26	−0.36 (−1.56 to 0.83)	0.55
Infection site, *n* (%)					
Pulmonary	225 (77.85%)	0.00 (Reference)		0.00 (Reference)	
Others*	64 (22.15%)	0.03 (−0.02 to 0.08)	0.31	−26.81 (−65.06 to 11.44)	0.17
Block agent, *n* (%)					
No	187 (64.71%)	0.00 (Reference)		0.00 (Reference)	
Yes	102 (35.29%)	−0.07 (−0.11 to −0.02)	< 0.001	−36.56 (−69.64 to −3.49)	0.03
Vasoactive agent, *n* (%)					
No	121(41.87%)	0.00 (Reference)		0.00 (Reference)	
Yes	168 (58.13%)	0.02 (−0.02 to 0.06)	0.39	−26.37 (−58.53 to 5.78)	0.11
HR (bpm), M (Q₁, Q₃)	109.00 (103.00,115.00)	0.00 (−0.00 to 0.00)	0.75	1.17 (−0.87 to 3.21)	0.26
MAP (mmHg), M (Q₁, Q₃)	70.00 (67.00,72.00)	0.00 (−0.00 to 0.01)	0.82	−1.38 (−5.52 to 2.75)	0.51
ScvO₂, M (Q₁, Q₃)	0.73 (0.71,0.75)	0.06 (−0.49 to 0.61)	0.83	458.03 (51.62 to 864.44)	0.03
Lac (mmol/L), M (Q₁, Q₃)	3.50 (2.60,4.40)	−0.00 (−0.01 to 0.01)	0.54	2.08 (−5.80 to 9.95)	0.61
CKMB (μg/L), M (Q₁, Q₃)	4.32 (3.23,6.34)	0.01 (−0.00 to 0.01)	0.26	0.93 (−5.70 to 7.56)	0.78
E/A, Mean ± SD	0.80 (0.23)	−0.06 (−0.15 to 0.04)	0.22	10.97 (−58.53 to 80.47)	0.76
LVEF (%), M (Q₁, Q₃)	56.84 (53.00,61.00)	−0.00 (−0.01 to 0.00)	0.46	−0.57 (−3.40 to 2.25)	0.69
SOFA, M (Q₁, Q₃)	8.00 (6.00,9.00)	−0.00 (−0.01 to 0.01)	0.42	3.96 (−3.04 to 10.95)	0.27
APACHE II, M (Q₁, Q₃)	18.00 (13.00,19.00)	0.00 (−0.00 to 0.00)	0.73	1.00 (−1.69 to 3.70)	0.47
ICU days, M (Q₁, Q₃)	7.00 (5.00,11.00)	0.00 (−0.00 to 0.01)	0.48	2.93 (−1.06 to 6.93)	0.15
Death in 28 days, *n* (%)					
No	217 (75.09%)	0.00 (Reference)		0.00 (Reference)	
Yes	72 (24.91%)	0.02 (−0.03 to 0.07)	0.50	13.32 (−23.49 to 50.13)	0.48

Abbreviations: APACHE II, Acute Physiology and Chronic Health Evaluation II; CI, confidence interval; CKMB, creatine kinase – MB isoenzyme; E/A, early diastolic velocity/atrial systolic velocity; HR, heart rate; ICU, intensive care unit; Lac, lactic acid; LVEF, left ventricular ejection fraction; MAP, mean arterial pressure; ScvO₂, central venous oxygen saturation; SOFA, Sequential Organ Failure Assessment.

* Abdominal cavity, urinary system, skin soft tissue, and so forth.

We further evaluated clinical outcomes. Neither 28‐day mortality (cTnI: *β* = 0.02, 95% CI: −0.03 to 0.07, *p* = 0.50; BNP: *β* = 13.32, 95% CI: −23.49 to 50.13, *p* = 0.48) nor ICU length of stay (cTnI: *β* = 0.00, 95% CI: −0.00 to 0.01, *p* = 0.48; BNP: *β* = 2.93, 95% CI: −1.06 to 6.93, *p* = 0.15) showed significant associations with biomarker levels (Table 2).

### 3.3 Relationship Between Long‐Term Oral β‐Blocker Use and cTnI and BNP

Table 3 presents the results of the regression analyses assessing the association between long‐term oral β‐blocker use and serum cTnI and BNP levels. In the unadjusted model (Model 1), β‐blocker use was significantly associated with lower cTnI (*β* = −0.07, 95% CI: −0.11 to −0.02, *p* < 0.001) and BNP levels (*β* = −36.56, 95% CI: −69.64 to −3.49, *p* = 0.03). These associations remained robust after adjustment for gender and age in Model 2 (cTnI: *β* = −0.07, 95% CI: −0.11 to −0.02, *p* < 0.001; BNP: *β* = −38.28, 95% CI: −71.77 to −4.79, *p* = 0.03) and after full adjustment for all potential confounders in Model 3 (cTnI: *β* = −0.06, 95% CI: −0.11 to −0.02, *p* < 0.001; BNP: *β* = −36.36, 95% CI: −71.45 to −1.26, *p* = 0.04).

**Table 3:** Multivariate regression analysis of the association between β‐blocker use and cardiac biomarkers in patients with septic myocardial injury.

Variables		Model 1		Model 2		Model 3	
		*β* (95% CI)	*p* value	*β* (95% CI)	*p* value	*β* (95% CI)	*p* value
cTnI (μg/L)	Block						
	No	0.00 (Reference)		0.00 (Reference)		0.00 (Reference)	
	Yes	−0.07 (−0.11 to −0.02)	< 0.001	−0.07 (−0.11 to −0.02)	< 0.001	−0.06 (−0.11 to −0.02)	< 0.001
BNP (ng/L)	Block						
	No	0.00 (Reference)		0.00 (Reference)		0.00 (Reference)	
	Yes	−36.56 (−69.64 to −3.49)	0.03	−38.28 (−71.77 to −4.79)	0.03	−36.36 (−71.45 to −1.26)	0.04

*Note:* Model 1: Crude; Model 2: Adjust: Gender, age; Model 3: Adjust all: Gender, age, infection site, vasoactive agent, HR, MAP, ScvO₂, Lac, CKMB, E/A, LVEF, SOFA, APACHE II, ICU days, death in 28 days.

Abbreviations: APACHE II, Acute Physiology and Chronic Health Evaluation II; BNP, brain natriuretic peptide; CI, confidence interval; CKMB, creatine kinase – MB isoenzyme; cTnI, cardiac troponin I; E/A, early diastolic velocity/atrial systolic velocity; HR, heart rate; ICU, intensive care unit; Lac, lactic acid; LVEF, left ventricular ejection fraction; MAP, mean arterial pressure; ScvO₂, central venous oxygen saturation; SOFA, Sequential Organ Failure Assessment.

### 3.4 Subgroup Analysis

To explore potential heterogeneity in the association between β‐blocker use and biomarker levels, subgroup analyses were performed, and the results are presented in Table 4. A more pronounced reduction in both cTnI and BNP levels was observed among patients younger than 60 years. Additionally, in patients with pulmonary infections, β‐blocker use was associated with a greater decrease in cTnI. In contrast, the associations across other subgroups, including gender, vasopressor use, and heart rate categories, remained largely consistent, reinforcing the overall correlation between β‐blocker therapy and reduced cTnI and BNP levels.

**Table 4:** Subgroup analysis of the association between β‐blocker use and cardiac biomarkers in patients with septic myocardial injury.

Variables	*n* (%)	*β* (95% CI)		*p* for interaction	
		cTnI	BNP	cTnI	BNP
All patients	289 (100.00)	−0.07 (−0.11 to −0.02)	−36.56 (−69.64 to −3.49)		
Gender				0.73	0.59
Male	151 (52.25)	−0.08 (−0.14 to −0.02)	−46.32 (−93.14 to 0.51)		
Female	138 (47.75)	−0.06 (−0.13 to 0.00)	−27.97 (−75.56 to 19.61)		
Age custom				0.52	0.56
< 60	40 (13.84)	−0.10 (−0.20 to −0.00)	−60.93 (−149.75 to 27.89)		
≥ 60	249 (86.16)	−0.06 (−0.11 to −0.01)	−33.14 (−68.95 to 2.67)		
Infection site				0.04	0.03
Pulmonary	225 (77.85)	−0.09 (−0.14 to −0.04)	−19.72 (−55.68 to 16.24)		
Others*	64 (22.15)	0.03 (−0.07 to 0.13)	−110.52 (−189.08 to −31.96)		
Vasoactive agent				0.71	0.35
No	121 (41.87)	−0.08 (−0.15 to −0.01)	−16.37 (−69.46 to 36.72)		
Yes	168 (58.13)	−0.06 (−0.12 to −0.01)	−48.65 (−90.75 to −6.55)		
HR (bpm)				0.15	0.08
89–104	93 (32.18)	−0.03 (−0.11 to 0.05)	9.79 (−43.36 to 62.94)		
105–112	97 (33.56)	−0.05 (−0.12 to 0.03)	−81.64 (−140.35 to −22.92)		
113–131	99 (34.26)	−0.14 (−0.22 to −0.06)	−34.50 (−98.07 to 29.07)		
MAP (mmHg)				0.31	0.92
65–67	73 (25.26)	−0.02 (−0.12 to 0.07)	−22.34 (−94.55 to 49.86)		
68–71	118 (40.83)	−0.11 (−0.17 to −0.04)	−35.73 (−88.06 to 16.59)		
72–80	98 (33.91)	−0.05 (−0.13 to 0.03)	−41.36 (−94.20 to 11.47)		
ScvO₂				0.87	0.23
0.61–0.70	57 (19.72)	−0.08 (−0.18 to 0.01)	−55.35 (−121.85 to 11.16)		
0.71–0.73	103 (35.64)	−0.05 (−0.12 to 0.02)	0.91 (−51.79 to 53.60)		
0.74–0.82	129 (44.64)	−0.06 (−0.13 to 0.01)	−59.80 (−113.89 to −5.71)		
Lac (mmol/L)				0.82	0.70
1.00–2.70	96 (33.22)	−0.05 (−0.13 to 0.02)	−24.97 (−83.29 to 33.35)		
2.80–4.00	97 (33.56)	−0.06 (−0.15 to 0.03)	−28.10 (−82.56 to 26.37)		
4.10–15.5	96 (33.22)	−0.09 (−0.15 to −0.02)	−56.44 (−115.33 to 2.45)		
CKMB (μg/L)				0.67	0.50
1.23–3.47	96 (33.22)	−0.07 (−0.15 to 0.01)	−63.58 (−124.40 to −2.77)		
3.49–5.41	96 (33.22)	−0.09 (−0.17 to −0.01)	−17.44 (−75.16 to 40.28)		
5.42–15.30	97 (33.56)	−0.04 (−0.11 to 0.03)	−27.97 (−81.93 to 25.99)		
E/A				0.36	0.49
0.30–0.68	94 (32.53)	−0.03 (−0.12 to 0.06)	−11.46 (−76.36 to 53.44)		
0.69–0.89	98 (33.91)	−0.11 (−0.18 to −0.04)	−31.09 (−83.74 to 21.56)		
0.90–1.54	97 (33.56)	−0.05 (−0.12 to 0.02)	−61.35 (−117.94 to −4.76)		
LVEF (%)				0.54	0.06
43.00–53.00	85 (29.41)	−0.03 (−0.11 to 0.06)	−95.09 (−155.35 to −34.82)		
54.00–58.81	103 (35.64)	−0.08 (−0.16 to −0.01)	3.49 (−54.24 to 61.21)		
59.00–71.00	101 (34.95)	−0.08 (−0.15 to −0.01)	−28.95 (−82.37 to 24.48)		
SOFA				0.59	0.79
2.00–5.00	66 (22.84)	−0.07 (−0.17 to 0.04)	−13.56 (−107.47 to 80.34)		
6.00–8.00	124 (42.91)	−0.04 (−0.10 to 0.02)	−47.26 (−92.44 to −2.08)		
9.00–14.00	99 (34.26)	−0.09 (−0.17 to −0.01)	−41.02 (−97.57 to 15.53)		
APACHE II				0.56	0.42
9.00–14.00	87 (30.10)	−0.10 (−0.17 to −0.03)	−26.86 (−81.62 to 27.90)		
15.00–18.00	97 (33.56)	−0.06 (−0.14 to 0.02)	−65.24 (−123.21 to −7.27)		
19.00–35.00	105 (36.33)	−0.04 (−0.12 to 0.04)	−12.56 (−73.11 to 47.99)		
ICU days				0.88	0.89
2.00–5.00	89 (30.80)	−0.07 (−0.15 to 0.01)	−27.39 (−90.41 to 35.63)		
6.00–8.00	84 (29.07)	−0.05 (−0.14 to 0.04)	−36.91 (−94.74 to 20.92)		
9.00–22.00	116 (40.14)	−0.08 (−0.14 to −0.01)	−46.90 (−98.83 to 5.03)		
Death in 28 days				0.61	0.84
No	217 (75.09)	−0.07 (−0.12 to −0.02)	−34.38 (−71.69 to 2.94)		
Yes	72 (24.91)	−0.05 (−0.15 to 0.05)	−42.29 (−113.81 to 29.23)		

*Note:* The above model adjusted for gender, age, infection site, vasoactive agent, HR, MAP, ScvO₂, Lac, CKMB, E/A, LVEF, SOFA, APACHE II, ICU days, and death in 28 days; in each case, the model is not adjusted for the stratification variable.

Abbreviations: APACHE II, Acute Physiology and Chronic Health Evaluation II; BNP, brain natriuretic peptide; CI, confidence interval; CKMB, creatine kinase – MB isoenzyme; cTnI, cardiac troponin I; E/A, early diastolic velocity/atrial systolic velocity; HR, heart rate; ICU, intensive care unit; Lac, lactic acid; LVEF, left ventricular ejection fraction; MAP, mean arterial pressure; ScvO₂, central venous oxygen saturation; SOFA, Sequential Organ Failure Assessment.

* Abdominal cavity, urinary system, skin soft tissue, and so forth.

## 4 Discussion

This retrospective single‐center study established a significant correlation between extended oral β‐blocker usage and decreased levels of serum cTnI and BNP in individuals with septic myocardial injury. This correlation persisted even after accounting for various confounding factors such as age, site of infection, and disease severity. Subgroup analysis indicated a more pronounced impact among patients younger than 60 years old and those with pulmonary infections, highlighting diverse advantages of β‐blockers within distinct patient cohorts.

The precise collection of all plasma indices within a uniform 24‐h window is crucial to accurately reflect the baseline condition of sepsis patients, especially those on long‐term β‐blocker therapy [5]. This approach allows for the measurement of stable plasma concentrations of β‐blockers in individuals using them regularly for at least 3 months, thereby reducing the impact of acute hospital interventions that could otherwise affect biomarker levels post‐admission [5]. This temporal consistency enhances the robustness of our analysis by distinguishing the chronic influence of β‐blockade on myocardial injury markers from transient changes caused by sepsis treatment or medication modifications during hospitalization [7]. Simultaneously measuring all pertinent plasma indices within the same 24‐h timeframe also effectively controls for confounding variables in multivariate models, thereby bolstering the credibility of our results regarding the independent correlation between β‐blockers and decreased cTnI/BNP levels [6].

Our results support the well‐documented myocardial protective properties of β‐blockers in cardiovascular conditions [14]. Previous research in heart failure and acute coronary syndrome has demonstrated that β‐blockers can mitigate myocardial damage by inhibiting excessive sympathetic activation, decreasing myocardial oxygen consumption, and reducing oxidative stress [15]. In the context of sepsis, uncontrolled activation of the sympathetic nervous system results in an overload of catecholamines, leading to myocardial calcium overload, mitochondrial dysfunction, and apoptosis through stimulation of β1‐adrenergic receptors [16]. The decreased levels of cTnI and BNP observed in individuals using β‐blockers suggest that these medications may alleviate sepsis‐induced myocardial cellular damage by blocking these detrimental pathways. It is important to note that our study specifically focuses on septic myocardial injury, as indicated by elevated biomarkers (cTnI/BNP), rather than septic cardiomyopathy, which necessitates evidence of cardiac dysfunction [17]. This targeted approach is in line with the clinical requirement for swift bedside evaluation and contributes novel insights into the potential early myocardial protective effects of β‐blockers in the setting of sepsis [18].

The protective effects of β‐blockers may be attributed to several mechanisms. First, by inhibiting Sympathetic Toxicity: Blocking cardiac β1 receptors reduces norepinephrine‐induced calcium overload and oxidative stress, thereby lowering the risk of myocardial cell apoptosis [19]. The observed significantly lower heart rate in the β‐blocker group (105 vs. 111 bpm, *p* < 0.001) indicates that heart rate regulation plays a crucial role in mediating their beneficial effects. Second, through anti‐inflammatory and immunomodulatory effects: β‐blockers suppress the nuclear factor‐κB pathway, leading to decreased release of pro‐inflammatory cytokines (e.g., TNF‐α, IL‐6) and alleviating sepsis‐induced cytokine storm‐mediated myocardial injury [20]. Additionally, β‐blockers contribute to the improvement in ventricular remodeling markers: The decrease in BNP, a marker of ventricular wall stress, may signify the role of β‐blockers in inhibiting myocardial fibrosis and retarding ventricular remodeling [21]. While this study did not directly evaluate cardiac function, the BNP alterations suggest potential protective effects on ventricular structure. Univariate analysis revealed a significant positive correlation between ScvO₂ and BNP (*β* = 458.03, *p* = 0.03), indicating a possible confounding effect of venous congestion. Despite the absence of a group difference in ScvO₂ (*p* = 0.12), this variable was included in multivariate models to isolate the independent impact of β‐blockers on BNP, ensuring that the outcomes were not influenced by hemodynamic factors associated with venous return [5].

Our results support the well‐documented myocardial‐protective properties of β‐blockers in cardiovascular conditions [14]. Prior heart failure and acute coronary syndrome studies have shown that β‐blockers mitigate myocardial damage via inhibition of excessive sympathetic activation, reduction of myocardial oxygen consumption, and alleviation of oxidative stress [15]. In sepsis, unchecked sympathetic stimulation leads to catecholamine overload, myocardial calcium imbalance, mitochondrial dysfunction, and apoptosis mediated by β₁‐adrenergic receptors [16]. The lower cTnI and BNP levels observed in β‐blocker users suggest that these medications may attenuate sepsis‐induced myocardial injury through the blockade of these harmful adrenergic pathways. Importantly, our focus here is on septic myocardial injury—indicated by elevated biomarkers—rather than septic cardiomyopathy, which requires demonstrable cardiac dysfunction [17]. This aligns with the clinical need for rapid bedside assessment and offers novel insight into the early myocardial protective effects of β‐blockers in sepsis [18].

Notably, while chronic β‐blocker use was associated with significant reductions in cardiac biomarkers, these improvements did not correspond to differences in 28‐day mortality or ICU length of stay in our cohort. This observation fits with emerging evidence emphasizing the prognostic relevance of peak rather than admission cTnI levels in sepsis. A recent ICU study (*n* = 333) demonstrated that elevated peak cTnI—unlike first‐day levels—was independently predictive of 28‐day mortality (adjusted HR: 2.33; 95% CI: 1.08–5.04; *p* = 0.03), underscoring the importance of dynamic biomarker monitoring in risk stratification [22, 23]. Similarly, a meta‐analysis indicated that although elevated admission cTnI/BNP are initially correlated with mortality, their significance often disappears after multivariate adjustment [24]. From a mechanistic standpoint, these findings may reflect pathophysiology rather than prognostic change. Septic cardiomyopathy is characterized by transient and reversible myocardial dysfunction, typically restoring normal function within 7–10 days [22]. Therefore, an early decrease in biomarkers like cTnI and BNP may indicate mitigation of acute myocardial injury rather than durable improvement in survival. Taken together, our data suggest that while β‐blockers may confer myocardial protection at the cellular level, the clinical relevance of biomarker improvements requires further validation. Several methodological factors may explain why biomarker reductions did not translate into improved clinical outcomes. First, the sample size (*n* = 289) may have been insufficient to detect modest differences in 28‐day mortality or ICU stay. Second, as an observational study, unmeasured confounding may have obscured true effects. Third, our short‐term evaluation may not capture the long‐term benefits of myocardial protection. Additionally, septic cardiomyopathy typically resolves within 7–10 days, suggesting that early biomarker changes may reflect transient rather than durable improvements. Adequately powered randomized trials with extended follow‐up are needed to establish clinical benefit. Future studies should incorporate serial biomarker measurements, early echocardiographic assessment of cardiac function, and extended follow‐up to determine whether early biomarker reductions can translate into meaningful improvements in sepsis outcomes.

These findings align with recent mechanistic insights into septic myocardial dysfunction. A 2024 narrative review describes septic cardiomyopathy as a global but reversible cardiac dysfunction—a window in which modulation of adrenergic and inflammatory pathways may yield benefit [22]. Moreover, echocardiography‐based evaluation of right ventricular dysfunction has been shown to predict in‐hospital mortality and lethal arrhythmias in sepsis, independent of left ventricular function [25]. Together, these observations underscore the broader cardiovascular implications of sepsis‐associated myocardial stress and lend further plausibility to β‐blockers' role in alleviating such injury, particularly through modulation of adrenergic overload and ventricular performance.

Several limitations should be noted in the interpretation of these findings. First, the retrospective design and single‐center nature of this study may introduce selection bias—certain baseline characteristics (e.g., stable cardiac status) may have influenced the decision to continue β‑blocker therapy. Even after excluding pre‐existing cardiovascular diseases and adjusting for measured covariates, unmeasured factors—including β‑blocker subtype or adherence—could still affect the observed associations [26]. Moreover, although baseline comorbidity and severity data were not systematically collected, it is plausible that patients on chronic β‑blocker therapy were more likely to have cardiovascular‐related risk factors such as hypertension or atrial fibrillation. This scenario exemplifies confounding by indication, where the clinical reasons for prescribing the drug are themselves associated with poorer prognosis, and such bias commonly pulls treatment‐outcome associations toward the null, leading to a potential underestimation of the true effect [27]. Despite this potential bias, we still observed statistically significant reductions in cTnI and BNP among β‑blocker users, suggesting that the findings may represent conservative estimates of a myocardial‐protective effect. However, residual and unmeasured confounding—capable of biasing results in either direction—cannot be completely ruled out [28]. Second, the demographic composition was heavily skewed toward elderly patients (mean age 73 years) and those with pulmonary infections (77.85%), which may limit the generalizability of our findings to younger or non‐pulmonary sepsis populations. Third, the absence of serial echocardiographic assessments of systolic or diastolic function restricts conclusions on whether β‑blockers improve cardiac performance; rather, our focus remains on short‐term changes in myocardial injury biomarkers [29]. An important limitation is the absence of data on concomitant cardiovascular medications (e.g., ACEI/ARB, statins, calcium channel blockers), which are commonly co‐prescribed with β‐blockers and may independently influence cardiac biomarkers. This key missing confounding factor limits our ability to isolate the independent effect of β‐blocker therapy. Future studies should systematically collect medication data to better characterize the cardioprotective role of β‐blockers in septic myocardial injury.

Our study proposes that prolonged administration of oral β‐blockers may mitigate myocardial cellular damage in septic myocardial injury by dampening sympathetic overactivity, which may underlie the enhanced biomarker responses seen in younger and pulmonary‐infected subgroups. These results have implications for clinical management, suggesting that β‐blockers could be maintained in hemodynamically stable sepsis patients with an existing long‐term prescription to potentially confer myocardial protection [30, 31]. In cases of new‐onset sepsis, further investigation is warranted to ascertain the safety and optimal timing for initiating β‐blockers early. Subsequent multicenter, prospective randomized controlled trials should assess the effects of β‐blockers on clinical outcomes such as 28‐day mortality and duration of mechanical ventilation, while also investigating potential variations in efficacy between β1‐selective and nonselective β‐blockers as well as different dosages [16, 32]. Importantly, the analyses of 28‐day mortality and ICU length of stay were exploratory, and future multicenter studies with serial biomarker monitoring and functional assessments are warranted to clarify their prognostic value.

## 5 Conclusion

This retrospective cohort study found that chronic β‐blocker use was independently associated with lower cTnI and BNP levels in septic myocardial injury patients, suggesting cardioprotective effects. Subgroup analyses showed greater reductions in patients under 60 years and those with pulmonary infections; however, these findings are exploratory given limited sample sizes and require validation. These biomarker improvements did not translate into differences in 28‐day mortality or ICU stay. Our findings support continuing β‐blockers in hemodynamically stable septic patients with prior use, but prospective trials are needed to establish the clinical benefit.

## Author Contributions

All authors have read and approved the final version of the manuscript. Chun Yang and Zheng Yang had full access to all the data in this study and takes complete responsibility for the integrity of the data and the accuracy of the data analysis.

## Disclosure

The funder had no role in the study design; data collection, analysis, or interpretation; writing of the manuscript; or the decision to submit for publication.

## Conflicts of Interest

The authors declare no conflicts of interest.

## Transparency Statement

The lead authors, Zheng Yang and Chun Yang, affirm that this manuscript is an honest, accurate, and transparent account of the study being reported; that no important aspects of the study have been omitted; and that any discrepancies from the study as planned (and, if relevant, registered) have been explained.

## Data Availability

The data that support the findings of this study are available on request from the corresponding author. The data are not publicly available due to privacy or ethical restrictions.
